# Predictors of Sustained Response With Tofacitinib Therapy in Patients With Ulcerative Colitis

**DOI:** 10.1093/ibd/izab278

**Published:** 2021-12-27

**Authors:** William J Sandborn, Alessandro Armuzzi, Giuseppina Liguori, Peter M Irving, Ala I Sharara, Rajiv Mundayat, Nervin Lawendy, John C Woolcott, Silvio Danese

**Affiliations:** Division of Gastroenterology, University of California San Diego, La Jolla, CA, USA; IBD Unit, Fondazione Policlinico Universitario A. Gemelli IRCCS, Università Cattolica del Sacro Cuore, Rome, Italy; Pfizer Srl, Rome, Italy; IBD Unit, Guy’s and St Thomas’ Hospital, London, UK; Division of Gastroenterology, American University of Beirut Medical Center, Beirut, Lebanon; Pfizer Inc., New York, NY, USA; Pfizer Inc., Collegeville, PA, USA; Pfizer Inc., Collegeville, PA, USA; Gastroenterology and Endoscopy, IRCCS Ospedale San Raffaele and University Vita-Salute San Raffaele, Milan, Italy

**Keywords:** predictors of response, tofacitinib, ulcerative colitis

## Abstract

**Background:**

Tofacitinib is an oral, small molecule JAK inhibitor for the treatment of ulcerative colitis. We evaluate baseline characteristics as predictors of sustained response and remission in patients with ulcerative colitis receiving tofacitinib maintenance therapy.

**Methods:**

Patients with clinical response following OCTAVE Induction 1 and 2 entered OCTAVE Sustain and were rerandomized to receive tofacitinib 5 or 10 mg twice daily or placebo. Baseline characteristics were stratified by week 52 efficacy endpoints (remission, sustained remission, clinical response, sustained clinical response). Associations between baseline characteristics and efficacy endpoints were evaluated using logistic regression analyses.

**Results:**

Overall, 170 of 487 (34.9%) patients were in remission at week 52. In multivariable modeling, endoscopic subscore at baseline of OCTAVE Induction 1 and 2 (2 vs 3; odds ratio [OR], 1.60; 95% confidence interval [CI], 1.06-2.44]), partial Mayo score (<2 vs ≥2; OR, 1.92; 95% CI, 1.27-2.90), and age (per 10-years; OR, 1.19; 95% CI, 1.02-1.39) at baseline of OCTAVE Sustain (following 8 weeks’ tofacitinib induction therapy) were associated with higher odds of remission at week 52. Oral corticosteroid use (OR, 0.63; 95% CI, 0.42-0.96) and C-reactive protein (per unit; OR, 0.94; 95% CI, 0.89-0.99) at baseline of OCTAVE Sustain were associated with reduced likelihood of remission at week 52. In general, opposite associations were observed for time to loss of response.

**Conclusion:**

Patients with greater clinical improvement after 8 weeks of tofacitinib induction therapy are more likely to maintain response or remission with tofacitinib regardless of dose received during maintenance, highlighting the importance of a robust response to induction therapy.

## Introduction

Ulcerative colitis (UC) is a chronic disease of the colon, characterized by relapsing and remitting inflammation.^1^ Ulcerative colitis is a heterogenous disease in terms of severity, disease course, and response to therapy.^2^ Secondary loss of response can often occur with UC therapies, with randomized clinical trials of ustekinumab, vedolizumab, tofacitinib, and infliximab reporting loss of response rates of 30%-55% after maintenance therapy ranging from 44-54 weeks.^3–6^ Therefore, multiple treatments with different mechanisms of action are required.

In order to better characterize patient populations, optimize treatment, and minimize adverse events (AEs), there is a need to identify factors that are predictive of maintaining response to therapy.^2^ Finding a way to predict response to therapy will help clinicians better assess patients who are at risk of poor outcomes and who are most likely to benefit from specific therapies.

Prediction of response to UC therapy is complex and is influenced by multiple factors, including the environment and microbiome. Molecular studies have shown some evidence on predictors of response to vedolizumab, but these have been carried out in small numbers.^7^ Although there have been studies into predictors of primary response to induction therapy, there have been limited investigations into predictors of sustained response during maintenance therapy. Previous studies of sustained remission have mainly focused on tumor necrosis factor inhibitor (TNFi) therapies or been conducted in pediatric cohorts.^8,9^

Tofacitinib is an oral, small molecule Janus kinase inhibitor for the treatment of UC. The efficacy and safety of tofacitinib have been evaluated in patients with UC in three phase 3 studies (2 identical 8-week induction studies [OCTAVE Induction 1 and 2] and a 52-week maintenance study [OCTAVE Sustain]).^5^

Here, we report on baseline demographics and clinical characteristics (from the baseline of either induction or maintenance) as predictors of sustained response and remission in patients with moderately to severely active UC receiving tofacitinib maintenance therapy.

## Materials and Methods

### Patients and Study Design

The OCTAVE Induction 1 and 2 studies (NCT01465763 and NCT01458951) were 2 identical, 8-week studies, in which patients with moderately to severely active UC were randomized to receive tofacitinib 10 mg twice daily (BID) or placebo. Patients who completed OCTAVE Induction 1 and 2 and achieved clinical response (defined as a decrease from induction study baseline total Mayo score of ≥3 points and ≥30%, plus a decrease in rectal bleeding subscore of ≥1 point or an absolute rectal bleeding subscore of 0 or 1) with either tofacitinib or placebo were eligible to enter the 52-week maintenance study, OCTAVE Sustain (NCT01458574), where they were rerandomized to receive tofacitinib 10 mg BID, tofacitinib 5 mg BID, or placebo. Details of concomitant medications are provided in the Supplementary Material. Full details of OCTAVE Induction 1 and 2 and OCTAVE Sustain have been reported previously.^5^

### Efficacy Assessments

Efficacy endpoints were based on the Mayo score that includes measurements of stool frequency, rectal bleeding, endoscopic subscore, and Physician Global Assessment (PGA; each with a range of 0 to 3). Potential predictors were assessed based on the following efficacy endpoints in OCTAVE Sustain: remission at week 52, sustained remission at weeks 24 and 52, clinical response at week 52, sustained clinical response at weeks 24 and 52, and time to loss of response. Remission was defined as a total Mayo score of ≤2 with no individual subscore >1, and a rectal bleeding subscore of 0. Sustained remission was defined as remission at weeks 24 and 52 of OCTAVE Sustain. Sustained clinical response was defined as clinical response at weeks 24 and 52 of OCTAVE Sustain. Loss of response was defined by an increase in partial Mayo score (PMS) of ≥2 points from baseline in OCTAVE Sustain for 2 consecutive visits (at least 2 weeks apart), with an increase in rectal bleeding subscore of ≥1 from baseline of OCTAVE Sustain.

### Safety Assessments

Safety outcomes and laboratory value changes were assessed in all patients with nonmissing efficacy responses in OCTAVE Sustain. The incidence and severity of AEs, classified by the Medical Dictionary for Regulatory Activities, including AEs of special interest, were reported. Gastrointestinal perforations, malignancies (excluding nonmelanoma skin cancer [NMSC]), NMSC, and major adverse cardiovascular events (MACE) were independently adjudicated. Safety outcomes and laboratory value changes were stratified by tofacitinib dose received during OCTAVE Sustain and remission status at week 52.

### Statistical Analyses

Observed data were analyzed using the full analysis set, including all randomized patients who completed OCTAVE Induction 1 and 2, achieved clinical response, and were subsequently assigned to tofacitinib 10 mg BID, tofacitinib 5 mg BID, or placebo in OCTAVE Sustain. Baseline demographics and clinical characteristics were analyzed descriptively based on the achievement of efficacy endpoints at week 52 or at both weeks 24 and 52, and summarized by treatment group. The following characteristics were assessed at baseline of OCTAVE Induction 1 and 2: C-reactive protein (CRP; mg/L), albumin (g/dL), total Mayo score, extraintestinal manifestations (EIMs), prior TNFi exposure, and prior TNFi failure. The following characteristics were assessed at baseline of OCTAVE Sustain (following 8 weeks of tofacitinib induction therapy): age, gender, CRP (mg/L), total Mayo score, PMS, EIMs, and oral corticosteroid use.

Univariate regression analyses were performed to evaluate if any baseline demographics or clinical characteristics at baseline of OCTAVE Sustain and/or OCTAVE Induction 1 and 2 were associated with remission at week 52 or time to loss of response during OCTAVE Sustain. The full list of evaluated factors is provided in the Supplementary Material.

To assess the effect of various factors on the likelihood of achieving remission at week 52, multivariable logistic regression modeling was performed using a stepwise selection procedure using an entry criterion *P* value of .15 and a stay criterion *P* value of .05. To assess the effect of various factors on time to loss of response endpoint, a time to event analysis was carried out using multivariable Cox proportional hazards regression modeling. A stepwise selection process was implemented, with an entry criterion *P* value of .15 and a stay criterion *P* value of .05.

Based on the final model, odds ratios (ORs) and 95% confidence intervals (CIs) from the multivariable logistic regression analysis were reported for factors associated with remission at week 52 of OCTAVE Sustain; and hazard ratios (HRs; 95% CI) from the multivariable Cox proportional hazards regression analysis were reported for factors associated with time to loss of response during OCTAVE Sustain.

These analyses were post hoc for exploratory purposes only, and no multiplicity adjustment was performed. For the univariate and multivariable analyses, nominal *P* values of ≤0.05 were considered significant.

### Ethical Considerations

All studies were conducted in compliance with the Declaration of Helsinki and the International Conference on Harmonization Good Clinical Practice Guidelines and were approved by the Institutional Review Boards and/or Independent Ethics Committees at each of the investigational centers participating in the studies, or a central Institutional Review Board. All patients provided written informed consent.

## Results

### Patients

The OCTAVE Sustain study included 593 patients who had initially received placebo, tofacitinib 10 mg BID, or tofacitinib 15 mg BID (a dose that was subsequently discontinued following a protocol amendment) induction therapy and had clinical response after 8 weeks before being rerandomized to receive placebo, tofacitinib 5 mg BID, or tofacitinib 10 mg BID.

Of the patients with available data for the respective efficacy outcomes, 34.9% of patients were in remission at week 52; 20.6% of patients achieved sustained remission at weeks 24 and 52; 54.2% of patients had a clinical response at week 52; and 51.5% of patients achieved sustained clinical response at weeks 24 and 52. The majority of patients who achieved these efficacy outcomes received tofacitinib 5 or 10 mg BID. Baseline demographics and disease characteristics were generally similar between treatment groups within respective efficacy outcomes (Tables 1-4).

**Table 1.** Baseline demographics and disease characteristics by week 52 remission status and treatment group.

	Tofacitinib 5 mg BID		Tofacitinib 10 mg BID		Placebo	
Remission at week 52	Yes N = 68	No N = 101	Yes N = 80	No N = 83	Yes N = 22	No N = 133
Age (years), mean (SD) ^a^	43.5 (14.9)	40.5 (12.5)	45.5 (15.0)	40.4 (13.0)	43.4 (12.5)	44.0 (14.0)
Age (<65 years), n (%)^a^	63 (92.6)	96 (95.0)	72 (90.0)	79 (95.2)	21 (95.5)	120 (90.2)
Male, n (%)^a^	40 (58.8)	49 (48.5)	40 (50.0)	54 (65.1)	11 (50.0)	84 (63.2)
CRP (mg/L) at baseline of OCTAVE Induction 1 and 2, mean (SD)^b^	7.6 (12.6)^c^	10.1 (19.4)	8.6 (13.9)^d^	8.5 (16.8)	10.4 (20.5)	10.5 (14.6)
CRP (mg/L) at baseline of OCTAVE Sustain, mean (SD)^a^	1.5 (2.0)	2.1 (3.5)	1.8 (3.8)	4.2 (7.3)	2.7 (3.4)	2.8 (5.0)
Albumin (g/dL), mean (SD)^b^	4.3 (0.4)	4.1 (0.4)	4.2 (0.5)	4.2 (0.4)	4.3 (0.3)	4.2 (0.4)
Total Mayo score, mean (SD)^b^	8.6 (1.5)	8.8 (1.4)	8.6 (1.5)	9.0 (1.5)	8.9 (1.3)	8.8 (1.3)
Total Mayo score <3, n (%)^a^	34 (50.0)	30 (29.7)	35 (43.8)	20 (24.1)	9 (40.9)	54 (40.6)
PMS <2, n (%)^a^	41 (60.3)	39 (38.6)	48 (60.0)	30 (36.1)	10 (45.5)	58 (43.6)
EIMs at baseline of OCTAVE Induction 1 and 2, n (%)^b^	13 (19.1)	31 (30.7)	19 (23.8)	19 (23.5)^e^	5 (22.7)	43 (32.3)
EIMs at baseline of OCTAVE Sustain, n (%)^a^	3 (4.4)	10 (9.9)	5 (6.3)	4 (4.9)^f^	1 (4.5)	18 (13.5)
Prior TNFi exposure, n (%)^b^	24 (35.3)	50 (49.5)	37 (46.3)	46 (55.4)	11 (50.0)	58 (43.6)
Prior TNFi failure, n (%)^b^	20 (29.4)	47 (46.5)	34 (42.5)	44 (53.0)	10 (45.5)	56 (42.1)
Oral corticosteroid use, n (%)^a^	29 (42.6)	60 (59.4)	25 (31.3)	39 (47.0)	11 (50.0)	60 (45.1)

Remission was defined as a total Mayo score of ≤2 with no individual subscore >1, and a rectal bleeding subscore of 0. Abbreviations: BID, twice daily; CRP, C-reactive protein; EIM, extraintestinal manifestation; N, number of patients in the specified category with nonmissing data; n, number of patients with the specified characteristic within the given category; PMS, partial Mayo score; SD, standard deviation; TNFi, tumor necrosis factor inhibitor.

Data at baseline of OCTAVE Sustain (following 8 weeks’ tofacitinib induction therapy).

Data at baseline of OCTAVE Induction 1 and 2.

N = 67.

N = 78.

N = 81.

N = 82.

**Table 2.** Baseline demographics and disease characteristics by sustained remission status and treatment group.

	Tofacitinib 5 mg BID		Tofacitinib 10 mg BID		Placebo	
Sustained remission at weeks 24 and 52	Yes N = 44	No N = 130	Yes N = 50	No N = 119	Yes N = 10	No N = 153
Age (years), mean (SD)^a^	41.9 (15.8)	41.9 (12.6)	44.2 (14.4)	42.3 (14.3)	38.0 (8.9)	44.3 (14.0)
Age (<65 years), n (%)^a^	40 (90.9)	124 (95.4)	46 (92.0)	110 (92.4)	10 (100.0)	138 (90.2)
Male, n (%)^a^	24 (54.5)	69 (53.1)	30 (60.0)	69 (58.0)	3 (30.0)	96 (62.7)
CRP (mg/L) at baseline of OCTAVE Induction 1 and 2, mean (SD)^b^	7.5 (13.0)	9.4 (17.9)^c^	7.7 (12.7)	9.5 (16.8)^d^	6.2 (8.1)	10.6 (15.6)
CRP (mg/L) at baseline of OCTAVE Sustain, mean (SD)^a^	1.7 (2.4)	2.0 (3.2)	1.4 (2.5)	3.7 (6.8)	3.2 (3.4)	3.1 (6.0)
Albumin (g/dL), mean (SD)^b^	4.3 (0.4)	4.2 (0.4)	4.2 (0.5)	4.2 (0.4)	4.3 (0.3)	4.2 (0.4)
Total Mayo score, mean (SD)^b^	8.6 (1.6)	8.7 (1.3)	8.5 (1.6)	9.0 (1.4)	8.9 (1.4)	8.9 (1.3)
Total Mayo score of <3, n (%)^a^	28 (63.6)	35 (26.9)	28 (56.0)	27 (22.7)	5 (50.0)	59 (38.6)
PMS <2, n (%)^a^	31 (70.5)	48 (36.9)	36 (72.0)	42 (35.3)	5 (50.0)	65 (42.5)
EIMs at baseline of OCTAVE Induction 1 and 2, n (%)^c^	9 (20.5)	37 (28.5)	10 (20.0)	30 (25.6)^e^	1 (10.0)	50 (32.7)
EIMs at baseline of OCTAVE Sustain, n (%)^a^	3 (6.8)	11 (8.5)	2 (4.0)	7 (5.9)^f^	0 (0.0)	20 (13.1)
Prior TNFi exposure, n (%)^b^	18 (40.9)	59 (45.4)	18 (36.0)	68 (57.1)	6 (60.0)	65 (42.5)
Prior TNFi failure, n (%)^b^	15 (34.1)	55 (42.3)	16 (32.0)	64 (53.8)	5 (50.0)	63 (41.2)
Oral corticosteroid use, n (%)^a^	16 (36.4)	73 (56.2)	14 (28.0)	54 (45.4)	5 (50.0)	70 (45.8)

Sustained remission was defined as remission at weeks 24 and 52 of OCTAVE Sustain. Remission was defined as a total Mayo score of ≤2 with no individual subscore >1, and a rectal bleeding subscore of 0. Abbreviations: BID, twice daily; CRP, C-reactive protein; EIM, extraintestinal manifestation; N, number of patients in the specified category with nonmissing data; n, number of patients with the specified characteristic within the given category; PMS, partial Mayo score; SD, standard deviation; TNFi, tumor necrosis factor inhibitor.

Data at baseline of OCTAVE Sustain (following 8 weeks’ tofacitinib induction therapy).

Data at baseline of OCTAVE Induction 1 and 2.

N = 129.

N = 114.

N = 117.

N = 118.

**Table 3.** Baseline demographics and disease characteristics by week 52 clinical response status and treatment group.

	Tofacitinib 5 mg BID		Tofacitinib 10 mg BID		Placebo	
Clinical response at week 52	Yes N = 102	No N = 67	Yes N = 122	No N = 41	Yes N = 40	No N = 115
Age (years), mean (SD)^a^	42.8 (14.2)	40.0 (12.4)	44.8 (14.3)	37.1 (12.4)	44.3 (12.8)	43.7 (14.2)
Age (<65 years), n (%)^a^	95 (93.1)	64 (95.5)	111 (91.0)	40 (97.6)	37 (92.5)	104 (90.4)
Male, n (%)^a^	57 (55.9)	32 (47.8)	67 (54.9)	27 (65.9)	23 (57.5)	72 (62.6)
CRP (mg/L) at baseline of OCTAVE Induction 1 and 2, mean (SD)^b^	9.4 (19.0)^c^	8.6 (13.6)	8.6 (16.0)^d^	8.2 (13.4)^e^	10.9 (19.0)	10.3 (14.2)
CRP (mg/L) at baseline of OCTAVE Sustain, mean (SD)^a^	1.8 (2.6)	1.9 (3.6)	2.6 (5.5)	4.2 (7.3)	2.4 (3.1)	2.9 (5.3)
Albumin (g/dL), mean (SD)^b^	4.2 (0.4)	4.1 (0.4)	4.2 (0.4)	4.1 (0.3)	4.2 (0.4)	4.2 (0.4)
Total Mayo score, mean (SD)^b^	8.7 (1.4)	8.8 (1.4)	8.7 (1.5)	9.1 (1.5)	9.0 (1.3)	8.8 (1.3)
Total Mayo score of <3, n (%)^a^	44 (43.1)	20 (29.9)	44 (36.1)	11 (26.8)	14 (35.0)	49 (42.6)
PMS <2, n (%)^a^	55 (53.9)	25 (37.3)	64 (52.5)	14 (34.1)	19 (47.5)	49 (42.6)
EIMs at baseline of OCTAVE Induction 1 and 2, n (%)^b^	24 (23.5)	20 (29.9)	30 (24.6)	8 (20.5)^f^	9 (22.5)	39 (33.9)
EIMs at baseline of OCTAVE Sustain, n (%)^a^	6 (5.9)	7 (10.4)	7 (5.7)	2 (5.0)^g^	3 (7.5)	16 (13.9)
Prior TNFi exposure, n (%)^b^	42 (41.2)	32 (47.8)	58 (47.5)	25 (61.0)	14 (35.0)	55 (47.8)
Prior TNFi failure, n (%)^b^	37 (36.3)	30 (44.8)	55 (45.1)	23 (56.1)	13 (32.5)	53 (46.1)
Oral corticosteroid use, n (%)^a^	42 (41.2)	47 (70.1)	39 (32.0)	25 (61.0)	18 (45.0)	53 (46.1)

Clinical response was defined as a decrease from induction study baseline total Mayo score of ≥3 points and ≥30%, plus a decrease in rectal bleeding subscore of ≥1 point or an absolute rectal bleeding subscore of 0 or 1. Abbreviations: BID, twice daily; CRP, C-reactive protein; EIM, extraintestinal manifestation; N, number of patients in the specified category with nonmissing data; n, number of patients with the specified characteristic within the given category; PMS, partial Mayo score; SD, standard deviation; TNFi, tumor necrosis factor inhibitor.

Data at baseline of OCTAVE Sustain (following 8 weeks’ tofacitinib induction therapy).

Data at baseline of OCTAVE Induction 1 and 2.

N = 101.

N = 119.

N = 38.

N = 39.

N = 40.

**Table 4.** Baseline demographics and disease characteristics by sustained clinical response status and treatment group.

	Tofacitinib 5 mg BID		Tofacitinib 10 mg BID		Placebo	
Sustained clinical response at weeks 24 and 52	Yes N = 97	No N = 72	Yes N = 117	No N = 45	Yes N = 38	No N = 120
Age (years), mean (SD)^a^	42.9 (14.2)	40.0 (12.5)	44.8 (14.4)	37.3 (12.5)	44.3 (13.1)	43.9 (14.2)
Age (<65 years), n (%)^a^	90 (92.8)	69 (95.8)	106 (90.6)	44 (97.8)	35 (92.1)	108 (90.0)
Male, n (%)^a^	53 (54.6)	36 (50.0)	65 (55.6)	29 (64.4)	22 (57.9)	74 (61.7)
CRP (mg/L) at baseline of OCTAVE Induction 1 and 2, mean (SD)^b^	9.2 (19.3)^c^	8.9 (13.5)	8.8 (16.2)^d^	8.1 (12.9)^e^	11.3 (19.4)	10.0 (14.0)
CRP (mg/L) at baseline of OCTAVE Sustain, mean (SD)^a^	1.8 (2.6)	2.1 (3.5)	2.6 (5.6)	4.0 (7.0)	2.5 (3.2)	3.2 (6.5)
Albumin (g/dL), mean (SD)^b^	4.3 (0.4)	4.1 (0.4)	4.2 (0.4)	4.2 (0.3)	4.2 (0.4)	4.2 (0.4)
Total Mayo score, mean (SD)^b^	8.6 (1.4)	8.8 (1.4)	8.8 (1.5)	9.0 (1.5)	9.0 (1.3)	8.8 (1.3)
Total Mayo score of <3, n (%)^a^	43 (44.3)	20 (27.8)	43 (36.8)	11 (24.4)	12 (31.6)	50 (41.7)
PMS <2, n (%)^a^	54 (55.7)	25 (34.7)	61 (52.1)	15 (33.3)	17 (44.7)	51 (42.5)
EIMs at baseline of OCTAVE Induction 1 and 2, n (%)^b^	22 (22.7)	23 (31.9)	27 (23.1)	9 (20.9)^f^	8 (21.1)	42 (35.0)
EIMs at baseline of OCTAVE Sustain, n (%)^a^	4 (4.1)	9 (12.5)	6 (5.1)	3 (6.8)^g^	3 (7.9)	17 (14.2)
Prior TNFi exposure, n (%)^b^	40 (41.2)	34 (47.2)	57 (48.7)	27 (60.0)	13 (34.2)	57 (47.5)
Prior TNFi failure, n (%)^b^	35 (36.1)	32 (44.4)	54 (46.2)	25 (55.6)	12 (31.6)	55 (45.8)
Oral corticosteroid use, n (%)^a^	40 (41.2)	48 (66.7)	37 (31.6)	27 (60.0)	16 (42.1)	56 (46.7)

Sustained clinical response was defined as clinical response at weeks 24 and 52 of OCTAVE Sustain. Clinical response was defined as a decrease from induction study baseline total Mayo score of ≥3 points and ≥30%, plus a decrease in rectal bleeding subscore of ≥1 point or an absolute rectal bleeding subscore of 0 or 1. Abbreviations: BID, twice daily; CRP, C-reactive protein; EIM, extraintestinal manifestation; N, number of patients in the specified category with nonmissing data; n, number of patients with the specified characteristic within the given category; PMS, partial Mayo score; SD, standard deviation; TNFi, tumor necrosis factor inhibitor.

Data at baseline of OCTAVE Sustain (following 8 weeks’ tofacitinib induction therapy).

Data at baseline of OCTAVE Induction 1 and 2.

N = 96.

N = 115.

N = 42.

N = 43.

N = 44.

### Efficacy

#### Clinical characteristics at baseline of OCTAVE Induction 1 and 2 that may predict efficacy outcomes with maintenance therapy

In the group receiving tofacitinib 5 mg BID, prior TNFi failure was observed in numerically lower proportions of patients who achieved remission (29.4%), sustained remission (34.1%), clinical response (36.3%), or sustained clinical response (36.1%) at week 52 compared with those who failed to achieve these endpoints (46.5%, 42.3%, 44.8%, and 44.4% of patients, respectively; Tables 1-4). Similar trends were observed in patients who received tofacitinib 10 mg BID (Tables 1-4).

#### Clinical characteristics at baseline of OCTAVE Sustain that may predict efficacy outcomes with maintenance therapy

In the group receiving tofacitinib 5 mg BID, PMS <2 at baseline of OCTAVE Sustain (following 8 weeks of tofacitinib induction therapy) was observed in numerically higher proportions of patients who achieved remission (60.3%), sustained remission (70.5%), clinical response (53.9%), or sustained clinical response (55.7%) at week 52 compared with those who did not achieve these endpoints (38.6%, 36.9%, 37.3%, and 34.7% of patients, respectively; Tables 1-4).

Similarly, total Mayo score of <3 at baseline of OCTAVE Sustain was observed in numerically higher proportions of patients in the group receiving tofacitinib 5 mg BID who achieved remission (50.0%), sustained remission (63.6%), clinical response (43.1%), or sustained clinical response (44.3%) at week 52 compared with those who did not achieve these endpoints (29.7%, 26.9%, 29.9%, and 27.8% of patients, respectively; Tables 1-4).

In the group receiving tofacitinib 5 mg BID, oral corticosteroid use at baseline of OCTAVE Sustain was observed in numerically lower proportions of patients who achieved remission (42.6%), sustained remission (36.4%), clinical response (41.2%), or sustained clinical response (41.2%) at week 52 compared with those who failed to achieve these endpoints (59.4%, 56.2%, 70.1%, and 66.7% of patients, respectively; Tables 1-4).

Similar trends were observed in patients who received tofacitinib 10 mg BID (Tables 1-4). Clinical characteristics among patients who received placebo during OCTAVE Sustain are shown in Tables 1-4.

#### Association between baseline factors and achievement of remission at week 52

**Figure 1.**
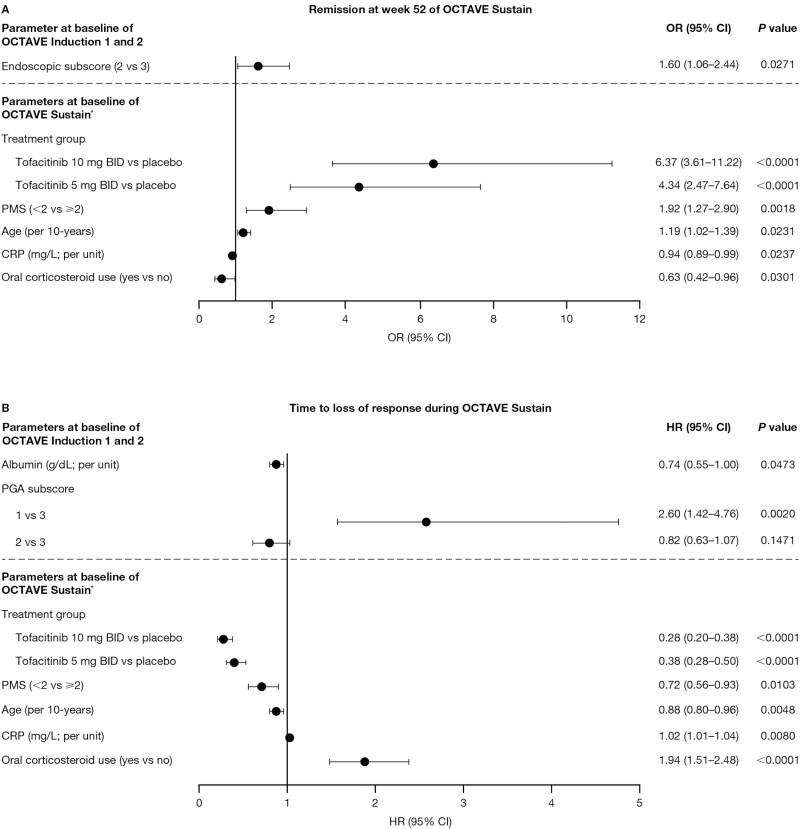
Multivariable analysis for factors associated with (A) remission at week 52 of OCTAVE Sustain and (B) time to loss of response during OCTAVE sustain. Stepwise selection (stay criterion at 0.05) was used to analyze the significant factors (*P* < .10) from the univariate analysis associated with (A) remission at week 52 of OCTAVE Sustain and (B) time to loss of response during OCTAVE Sustain. ^∗^Following 8 weeks’ tofacitinib induction therapy. Abbreviations: BID, twice daily; CI, confidence interval; CRP, C-reactive protein; HR, hazard ratio; OR, odds ratio; PGA, Physician Global Assessment; PMS, partial Mayo score.

Univariate logistic regression analysis for factors associated with remission at week 52 are shown in Supplementary Tables 1-3. In the multivariable model, lower endoscopic subscore at baseline of OCTAVE Induction 1 and 2 was associated with higher odds of remission at week 52 (2 vs 3; OR, 1.60; 95% CI, 1.06-2.44; Figure 1A). Both tofacitinib 10 mg BID vs placebo and tofacitinib 5 mg BID vs placebo received during OCTAVE Sustain were significantly associated with higher odds of remission at week 52, with a higher likelihood of patients achieving remission in the group receiving 10 mg BID vs placebo compared with patients in the group receiving 5 mg BID vs placebo (OR, 6.37; 95% CI, 3.61-11.22 and OR, 4.34; 95% CI, 2.47-7.64, respectively; Figure 1A). Lower PMS (<2 vs ≥2; OR, 1.92; 95% CI, 1.27-2.90) and older age (continuous; per 10-years; OR, 1.19; 95% CI, 1.02-1.39) at baseline of OCTAVE Sustain were also associated with higher odds of remission at week 52 (Figure 1A).

Conversely, factors associated with a reduced likelihood of remission at week 52 were oral corticosteroid use (OR, 0.63; 95% CI, 0.42-0.96) and higher CRP (per unit; OR, 0.94; 95% CI, 0.89-0.99) at baseline of OCTAVE Sustain (Figure 1A).

**Figure 2.**
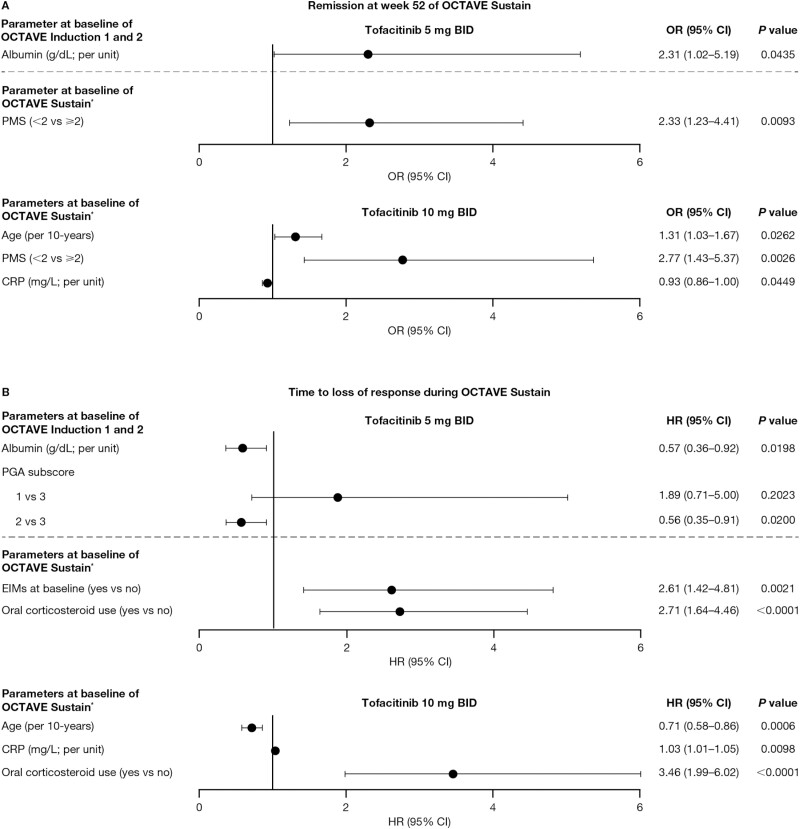
Multivariable analysis by tofacitinib dose group for factors associated with (A) remission at week 52 of OCTAVE Sustain and (B) time to loss of response during OCTAVE Sustain. Stepwise selection (stay criterion at 0.05) was used to analyze the significant factors (*P* < .10) from the univariate analysis associated with (A) remission at week 52 of OCTAVE Sustain and (B) time to loss of response during OCTAVE Sustain. ^∗^Following 8 weeks’ tofacitinib induction therapy. Abbreviations: BID, twice daily; CI, confidence interval; CRP, C-reactive protein; EIM, extraintestinal manifestation; HR, hazard ratio; OR, odds ratio; PGA, Physician Global Assessment; PMS, partial Mayo score.

When analyzed by dose group, higher albumin (continuous; per unit) at baseline of OCTAVE Induction 1 and 2 was associated with higher odds of remission at week 52 among patients receiving 5 mg of tofacitinib BID—but not 10 mg of tofacitinib BID. Older age (continuous; per 10-years) was associated with higher odds, and higher CRP (continuous; per unit) at baseline of OCTAVE Sustain was associated with lower odds of remission at week 52 among patients receiving tofacitinib 10 mg BID, but not tofacitinib 5 mg BID (Figure 2A). Lower PMS (<2 vs ≥2) at baseline of OCTAVE Sustain was associated with higher odds of remission at week 52 among patients receiving tofacitinib 5 mg BID and tofacitinib 10 mg BID (Figure 2A).

A total of 462 (94.9%) patients had nonmissing data for all the factors included in the analysis of remission at week 52. Of these, 161 (34.8%) patients achieved remission at week 52, and 301 (65.2%) did not achieve remission.

#### Association between baseline factors and loss of response during OCTAVE Sustain

Univariate Cox proportional hazards regression analysis showing factors associated with time to loss of response during OCTAVE Sustain are shown in Supplementary Tables 4-6.

In the multivariable model, lower PGA subscore at baseline of OCTAVE Induction 1 and 2 was associated with an increased risk of loss of response during OCTAVE Sustain (1 vs 3; HR, 2.60; 95% CI, 1.42-4.76; Figure 1B).

Factors at baseline of OCTAVE Sustain that were significantly associated with increased risk of loss of response during OCTAVE Sustain were oral corticosteroid use (HR, 1.94; 95% CI, 1.51-2.48) and higher CRP (continuous; per unit; HR, 1.02; 95% CI, 1.01-1.04).

Conversely, factors associated with a reduced risk of loss of response were higher albumin at baseline of OCTAVE Induction 1 and 2 (continuous; per unit; HR, 0.74; 95% CI, 0.55-1.00), treatment with tofacitinib 5 or 10 mg BID maintenance therapy (vs placebo; HR, 0.38; 95% CI, 0.28-0.50 and HR, 0.28; 95% CI, 0.20-0.38, respectively), older age (continuous; per 10-years; HR, 0.88; 95% CI, 0.80-0.96), and lower PMS at baseline of OCTAVE Sustain (<2 vs ≥2; HR, 0.72; 95% CI, 0.56-0.93; Figure 1B).

When analyzed by dose group, higher albumin (continuous; per unit) and lower PGA subscore (2 vs 3) at baseline of OCTAVE Induction 1 and 2 were associated with a reduced risk, and the presence of EIMs at baseline of OCTAVE Sustain was associated with an increased risk of loss of response among patients receiving tofacitinib 5 mg BID, but not tofacitinib 10 mg BID. Older age (continuous; per 10-years) at baseline of OCTAVE Sustain was associated with a reduced risk, and higher CRP (continuous; per unit) at baseline of OCTAVE Sustain was associated with an increased risk of loss of response among patients receiving tofacitinib 10 mg BID, but not tofacitinib 5 mg BID (Figure 2B). Oral corticosteroid use at baseline of OCTAVE Sustain was associated with an increased risk of loss of response among patients receiving tofacitinib 5 mg BID and tofacitinib 10 mg BID.

### Safety

A summary of safety, AEs of special interest, and laboratory parameters stratified by remission status at week 52 and treatment group is presented in Supplementary Table 7.

The proportion of patients with AEs was generally similar, regardless of remission status (Supplementary Table 7). A numerically lower proportion of patients with remission discontinued due to AEs compared with patients without remission, regardless of treatment group (Supplementary Table 7).

Among patients receiving tofacitinib 10 mg BID or placebo, a numerically higher proportion of patients with remission at week 52 had infection AEs compared with patients without remission (Supplementary Table 7). This was not observed in patients receiving tofacitinib 5 mg BID, where a comparable number of infection AEs were observed between those with remission at week 52 and those without remission. No serious infections, gastrointestinal perforations, or malignancies (excluding NMSC) occurred in any treatment group in OCTAVE Sustain (among patients with nonmissing efficacy responses). There were 2 cases of NMSC in the group of patients receiving tofacitinib 10 mg BID (1 of which occurred in a patient with remission at week 52) and 1 case in the placebo group in a patient without remission at week 52. There was 1 case of MACE in the group receiving tofacitinib 5 mg BID; the patient achieved remission at week 52. There was 1 case each of pulmonary embolism and deep vein thrombosis, both occurring in the placebo group in patients without remission at week 52. The proportions of patients with herpes zoster (nonserious and serious) were similar between patients, regardless of remission status, across treatment groups (Supplementary Table 7).

Among patients receiving tofacitinib 5 mg BID or tofacitinib 10 mg BID, a generally numerically higher proportion of patients with remission at week 52 had total cholesterol >1.3× upper limit of normal (ULN), triglycerides >1.3× ULN, low-density lipoprotein >1.2× ULN, and creatine kinase >2× ULN compared with patients without remission (Supplementary Table 7). The proportion of patients with high-density lipoprotein <0.8× lower limit of normal was low across treatment groups, regardless of remission status (Supplementary Table 7). Laboratory parameters were generally similar among patients receiving placebo, regardless of remission status (Supplementary Table 7).

## Discussion

This report presents post hoc analyses evaluating baseline demographics and clinical characteristics as potential predictors of achieving efficacy outcomes following 52 weeks of tofacitinib maintenance therapy. A deeper response to tofacitinib induction therapy and no oral corticosteroid use at baseline of OCTAVE Sustain, following 8 weeks of tofacitinib induction therapy, were key characteristics associated with remission and maintaining clinical response at week 52 compared with more severe disease and oral corticosteroid use. Partial Mayo score <2 at baseline of OCTAVE Sustain was significantly associated with an increased likelihood of achieving remission and a reduced risk of loss of response during OCTAVE Sustain vs PMS ≥2. Lower endoscopic subscore (moderate vs severe disease) at baseline of OCTAVE Induction 1 and 2 was also associated with remission at week 52. Higher albumin at baseline of OCTAVE Induction 1 and 2 was associated with reduced risk of loss of response during OCTAVE Sustain. Conversely, higher CRP and corticosteroid use at baseline of OCTAVE Sustain were significantly associated with an increased risk of loss of response and a reduced likelihood of remission at week 52. Furthermore, lower PGA subscore (1 vs 3) at baseline of OCTAVE Induction 1 and 2 was associated with loss of response during OCTAVE Sustain. Physician Global Assessment subscore is a subjective measure of efficacy and a recognized Mayo score limitation.^10^

When analyzed by dose group, higher albumin at baseline of OCTAVE Induction 1 and 2 was associated with an increased likelihood of achieving remission and a reduced risk of loss of response among patients receiving tofacitinib 5 mg BID, but not tofacitinib 10 mg BID. The albumin level at baseline of OCTAVE Induction 1 and 2 was relatively high across treatment groups, being at the upper end of the normal range for the general population. A previous analysis of data from the tofacitinib UC clinical program showed that tofacitinib clearance was not related to albumin concentration, and after accounting for the significant effect of baseline Mayo score in the multivariable analysis, baseline albumin had no effect on efficacy endpoints in OCTAVE Induction 1 and 2 and OCTAVE Sustain. Therefore, baseline albumin may not be useful in determining optimal tofacitinib dose in patients with UC, beyond its association with disease severity as determined by the total Mayo score.^11^ The presence of EIMs at baseline of OCTAVE Sustain was associated with increased risk of loss of response among patients receiving tofacitinib 5 mg BID, but not tofacitinib 10 mg BID. Although sample sizes were small, when analyzed by dose group, tofacitinib 10 mg BID may be more efficacious in maintaining response in more complex UC (eg, UC with EIMs) compared with tofacitinib 5 mg BID. This is supported by recent findings showing that, in patients with EIMs at baseline, the proportion of patients achieving remission at week 52 of OCTAVE Sustain was higher among patients receiving tofacitinib 10 mg BID vs 5 mg BID (41.3% vs 24.1%, respectively).^12^ Higher CRP at baseline of OCTAVE Sustain was associated with a reduced likelihood of achieving remission and increased risk of loss of response among patients receiving tofacitinib 10 mg BID, but not tofacitinib 5 mg BID. This result could be influenced by small sample size in both dose groups and a large standard deviation in the group receiving tofacitinib 10 mg BID not in remission (mean CRP [mg/L] 4.2, standard deviation 7.3). The opposite association was observed with increasing age; however, this finding could also have been influenced by small sample size in both dose groups.

Partial Mayo score <2 at baseline of OCTAVE Sustain was significantly associated with an increased likelihood of achieving remission and a reduced risk of loss of response during OCTAVE Sustain vs PMS ≥2. It is noted that the extent and severity of UC is best assessed by endoscopy, with the endoscopic subscore as a core component of the Mayo score.^1^ In a comparison between total Mayo score and the noninvasive PMS, PMS has been found to perform as well as the total Mayo score in identifying patient-perceived clinical response.^13^ Therefore, PMS can be considered a practical measure of predicting which patients are likely to maintain response and achieve remission following 52 weeks of tofacitinib maintenance therapy.

Here, higher CRP at baseline of OCTAVE Sustain was associated with an increased risk of loss of response and a lower likelihood of achieving remission at week 52. Because CRP is an objective marker of inflammation, CRP levels have been observed to correlate with the extent of the disease.^14^ Consistent with this, higher CRP at baseline was independently associated with primary nonresponse to 8-week tofacitinib induction therapy in a recent real-world cohort study of patients with moderate to severe UC.^15^ Furthermore, in an observational study of patients with steroid-dependent UC treated with infliximab maintenance therapy, an endoscopic subscore of 3 (indicative of severe disease) at baseline and high CRP after infliximab induction therapy were independent predictors of colectomy.^16^

Oral corticosteroid use at baseline of OCTAVE Sustain was significantly associated with loss of response and lower odds of achieving remission at week 52 of tofacitinib treatment vs no corticosteroid use. Corticosteroid requirement is generally associated with poor UC prognosis; a meta-analysis looking at predictors of colectomy in patients with UC showed that the need for corticosteroids was associated with a higher risk of colectomy.^17^

Because active disease is a potential risk factor for AEs, it is important to find predictors of response that may reduce the risk of relapsing disease activity during maintenance therapy. The safety profile of tofacitinib 5 mg BID or tofacitinib 10 mg BID as maintenance therapy demonstrated no safety risks associated with remission status at week 52 of OCTAVE Sustain. Discontinuations due to AEs were generally less frequent among patients who achieved remission compared with those who did not. The safety data in these studies are consistent with the known safety profile of tofacitinib.^5^

A limitation of the current study is that these are post hoc analyses and the pooled studies were not designed, nor powered, to identify or evaluate predictors of response to maintenance treatment. Additional data interpretation is limited by small numbers. Although fecal calprotectin level has shown evidence of predicting sustained efficacy,^18^ this biomarker was not measured in the OCTAVE program and thus could not be included in this analysis. Furthermore, patients enrolled in OCTAVE Sustain were prior responders to 8-week treatment with tofacitinib 10 mg BID or placebo in OCTAVE Induction 1 and 2.

## Conclusions

In conclusion, the results of these analyses suggest that PMS, CRP, and corticosteroid use are all characteristics that could be evaluated after 8 weeks of tofacitinib induction therapy to provide some prediction as to whether patients are likely to achieve remission or lose response to tofacitinib maintenance therapy. Furthermore, a lower endoscopic subscore at baseline of induction therapy may be predictive of remission at week 52 of tofacitinib maintenance therapy. Overall, these results suggest that patients with a deeper response to tofacitinib induction therapy are more likely to maintain response or remission compared with patients with more severe UC, regardless of tofacitinib dose received during maintenance. Therefore, this highlights the importance of a robust response to induction therapy in the treatment of UC.

## Supplementary Material

izab278_suppl_Supplementary_Material

## Data Availability

Upon request, and subject to review, Pfizer will provide the data that support the findings of this study. Subject to certain criteria, conditions and exceptions, Pfizer may also provide access to the related individual anonymized participant data. See https://www.pfizer.com/science/clinical-trials/trial-data-and-results for more information.
