# Supplementary material for: Predictors of Sustained Response With Tofacitinib Therapy in Patients With Ulcerative Colitis
Source: Inflamm Bowel Dis. 2021 Dec 27;28(9):1338–47. doi: 10.1093/ibd/izab278 (PMC9434448; doi:10.1093/ibd/izab278)
Supplement: izab278_suppl_Supplementary_Material [file izab278_suppl_supplementary_material.pdf]

SUPPLEMENTARY DATA

Concomitant Medications.. 3

Logistic Regression Analysis 3

SUPPLEMENTARY TABLE 1. Univariate Logistic Regression Analysis For Factors Associated With Remission at Week 52 of OCTAVE Sustain Among All Patients With Non-Missing Efficacy Values in OCTAVE Sustain.. 5

SUPPLEMENTARY TABLE 2. Univariate Logistic Regression Analysis For Factors Associated With Remission at Week 52 of OCTAVE Sustain Among All Patients With Non-Missing Efficacy Values Who Received Tofacitinib 5 mg BID in OCTAVE Sustain............. 7

SUPPLEMENTARY TABLE 3. Univariate Logistic Regression Analysis For Factors Associated With Remission at Week 52 of OCTAVE Sustain Among All Patients With Non-Missing Efficacy Values Who Received Tofacitinib 10 mg BID in OCTAVE Sustain........... 8

SUPPLEMENTARY TABLE 4. Univariate Cox Proportional Hazards Regression Analysis For Factors Associated With Time to Loss of Response During OCTAVE Sustain Among All Patients With Non-Missing Efficacy Values in OCTAVE Sustain...................................... 10

SUPPLEMENTARY TABLE 5. Univariate Cox Proportional Hazards Regression Analysis For Factors Associated With Time to Loss of Response During OCTAVE Sustain Among All Patients With Non-Missing Efficacy Values Who Received Tofacitinib 5 mg BID in OCTAVE Sustain.. 12

SUPPLEMENTARY TABLE 6. Univariate Cox Proportional Hazards Regression Analysis For Factors Associated With Time to Loss of Response During OCTAVE Sustain Among All Patients With Non-Missing Efficacy Values Who Received Tofacitinib 10 mg BID in OCTAVE Sustain.. 14

SUPPLEMENTARY TABLE 7. Summary of Safety, AEs of Special Interest, and Laboratory Value Changes, By Week 52 Remission Status and Treatment Group 16

Concomitant Medications

Oral corticosteroids (prednisone-equivalent up to 25 mg/day or budesonide up to 9 mg/day) were permitted during the induction studies, provided that the dose remained stable for at least 2 weeks prior to baseline and throughout the induction study period. Corticosteroid tapering was mandatory at the beginning of OCTAVE Sustain. Prohibited concomitant therapies during OCTAVE Sustain included tumor necrosis factor inhibitors (TNFi) and immunomodulators.

Logistic Regression Analysis

The following variables were evaluated at baseline of OCTAVE Induction 1 and 2: albumin (g/dL) (continuous [per unit]; ≤3.5 vs >3.5 [remission at week 52 analysis] or <3.5 vs ≥3.5 [loss of response during OCTAVE Sustain analysis]), C-reactive protein (CRP; mg/L) (continuous [per unit]; ≤3 vs >3; ≤6 vs >6), disease duration (<6 vs ≥6 years), endoscopic subscore (2 vs 3), extent of disease, presence of extraintestinal manifestations (EIMs), prior TNFi exposure, prior TNFi failure, rectal bleeding subscore, stool frequency subscore, Physician Global Assessment subscore, and total Mayo score (per 1-point).

The following variables were evaluated at baseline of OCTAVE Sustain: age (continuous [per 10-years]; <30 vs ≥50; 30–<40 vs ≥50; 40–<50 vs ≥50; <65 vs ≥65), CRP (mg/L) (continuous [per unit]; ≤3 vs >3; ≤6 vs >6), gender, oral corticosteroid use, endoscopic improvement (defined as a Mayo endoscopic subscore of 0 or 1 and referred to as mucosal healing in the original OCTAVE protocols [Sandborn WJ, Su C, Sands BE, et al. Tofacitinib as induction and maintenance therapy for ulcerative colitis. N Engl J Med. 2017;376:1723–36]) from baseline to week 8 of OCTAVE Induction 1 and 2, presence of EIMs, race, remission status, partial Mayo score (<2 vs ≥2), and total Mayo score (<3 vs ≥3).

Tofacitinib dose received during OCTAVE Sustain was also evaluated (tofacitinib 10 mg twice daily vs placebo; tofacitinib 5 mg twice daily vs placebo).

SUPPLEMENTARY TABLE 1. Univariate Logistic Regression Analysis For Factors Associated With Remission at Week 52 of OCTAVE Sustain Among All Patients With Non-Missing Efficacy Values in OCTAVE Sustain

	OR (95% CI)	*P* value
Treatment[†]		<0.0001[‡]
Tofacitinib 10 mg BID vs placebo	5.83 (3.38–10.05)	
Tofacitinib 5 mg BID vs placebo	4.07 (2.36–7.02)	
Endoscopic subscore (2 vs 3)[†]	1.50 (1.03–2.18)	0.0354
Total Mayo score (<3 vs ≥3)[*]	1.74 (1.19–2.54)	0.0046
Partial Mayo score (<2 vs ≥2)[*]	2.09 (1.43–3.05)	0.0001
Remission status (yes vs no)[*]	1.64 (1.11–2.43)	0.0135
Extent of disease[‡]		0.0219[‡]
Left-sided colitis vs extensive colitis/pancolitis	1.29 (0.85–1.96)	
Proctosigmoiditis/proctitis vs extensive colitis/pancolitis	2.11 (1.24–3.58)	
CRP (mg/L)[*]		
CRP (per unit)	0.93 (0.88–0.99)	0.0140
CRP (≤3 vs >3 mg/L)	2.00 (1.23–3.25)	0.0053
CRP (≤6 vs >6 mg/L)	2.51 (1.23–5.13)	0.0116
Oral corticosteroid use (yes vs no)[*]	0.62 (0.42–0.90)	0.0121

Remission was defined as a total Mayo score of ≤2 with no individual subscore >1, and a rectal bleeding subscore of 0. The following factors were not significant ($P > 0.05$): albumin (g/dL) (continuous [per unit]; ≤3.5 vs >3.5), CRP (mg/L) (continuous [per unit]; ≤3 vs >3; ≤6 vs >6), disease duration (<6 vs ≥6 years), presence of EIM, prior TNFi exposure, prior TNFi failure, rectal bleeding subscore, stool frequency subscore, PGA subscore, total Mayo score (all at baseline of OCTAVE Induction 1 and 2); age (continuous [per 10-years]; <30 vs

≥50; 30–<40 vs ≥50; 40–<50 vs ≥50; <65 vs ≥65 years), gender, race (Asian vs other; Black vs other; White vs other), endoscopic improvement and presence of EIM (all at baseline of OCTAVE Sustain).

*Data at baseline of OCTAVE Sustain.

†Data at baseline of OCTAVE Induction 1 and 2.

‡Overall effect.

BID, twice daily; CI, confidence interval; CRP, C-reactive protein; EIM, extraintestinal manifestation; NA, not applicable; OR, odds ratio; PGA, Physician Global Assessment; TNFi, tumor necrosis factor inhibitor.

SUPPLEMENTARY TABLE 2. Univariate Logistic Regression Analysis For Factors Associated With Remission at Week 52 of OCTAVE Sustain Among All Patients With Non-Missing Efficacy Values Who Received Tofacitinib 5 mg BID in OCTAVE Sustain

	**OR (95% CI)**	***P* value**
Total Mayo score (<3 vs ≥3)*	2.37 (1.25–4.48)	0.0082
Partial Mayo score (<2 vs ≥2)*	2.41 (1.29–4.53)	0.0061
Remission status (yes vs no)*	2.06 (1.08–3.93)	0.0288
Oral corticosteroid use (yes vs no)*	0.51 (0.27–0.95)	0.0333
Albumin (g/dL; per unit)†	2.41 (1.10–5.30)	0.0286
Prior TNFi failure status (yes vs no)†	0.48 (0.25–0.92)	0.0268

Remission was defined as a total Mayo score of ≤2 with no individual subscore >1, and a rectal bleeding subscore of 0. The following factors were not significant (*P* > 0.05): albumin (g/dL) (≤3.5 vs >3.5), CRP (mg/L) (continuous [per unit]; ≤3 vs >3; ≤6 vs >6), disease duration (<6 vs ≥6 years), endoscopic subscore (2 vs 3), extent of disease, presence of EIM, prior TNFi exposure, rectal bleeding subscore, stool frequency subscore, PGA subscore, total Mayo score (all at baseline of OCTAVE Induction 1 and 2); age (continuous [per 10-years]; <30 vs ≥50; 30–<40 vs ≥50; 40–<50 vs ≥50; <65 vs ≥65 years), CRP (mg/L) (continuous [per unit]; ≤3 vs >3; ≤6 vs >6), gender, race (Asian vs other; Black vs other; White vs other), endoscopic improvement and presence of EIM (all at baseline of OCTAVE Sustain).

*Data at baseline of OCTAVE Sustain.

†Data at baseline of OCTAVE Induction 1 and 2.

BID, twice daily; CI, confidence interval; CRP, C-reactive protein; EIM, extraintestinal manifestation; OR, odds ratio; PGA, Physician Global Assessment; TNFi, tumor necrosis factor inhibitor.

SUPPLEMENTARY TABLE 3. Univariate Logistic Regression Analysis For Factors Associated With Remission at Week 52 of OCTAVE Sustain Among All Patients With Non-Missing Efficacy Values Who Received Tofacitinib 10 mg BID in OCTAVE Sustain

	**OR (95% CI)**	***P* value**
Total Mayo score (<3 vs ≥3)*	2.45 (1.25–4.79)	0.0087
Partial Mayo score (<2 vs ≥2)*	2.65 (1.41–4.99)	0.0025
Remission status (yes vs no)*	2.28 (1.15–4.55)	0.0188
Oral corticosteroid use (yes vs no)*	0.51 (0.27–0.97)	0.0408
Endoscopic improvement (yes vs no)*	2.05 (1.10–3.83)	0.0246
CRP (mg/L)*		
CRP (per unit)	0.91 (0.84–0.99)	0.0244
CRP (≤3 vs >3 mg/L)	3.80 (1.66–8.74)	0.0016
CRP (≤6 vs >6 mg/L)	4.54 (1.45–14.24)	0.0096
Age (per 10-years)*	1.30 (1.04–1.63)	0.0233
Age (categorical)*,†		0.0089‡
Age (30–<40 vs ≥50 years)	0.35 (0.16–0.81)	0.0142
Age (40–<50 vs ≥50 years)	0.22 (0.09–0.56)	0.0014

Remission was defined as a total Mayo score ≤2 with no individual subscore >1, and a rectal bleeding subscore of 0. The following factors were not significant (*P* > 0.05): albumin (g/dL) (continuous [per unit]; ≤3.5 vs >3.5), CRP (mg/L) (continuous [per unit]; ≤3 vs >3; ≤6 vs >6), disease duration (<6 vs ≥6 years), endoscopic subscore (2 vs 3), extent of disease, presence of EIM, prior TNFi exposure, prior TNFi failure, rectal bleeding subscore, stool frequency subscore, PGA subscore, total Mayo score (all at baseline of OCTAVE Induction 1 and 2); age (<65 vs ≥65 years), gender, race (Asian vs other; Black vs other; White vs other), and presence of EIM (all at baseline of OCTAVE Sustain).

*Data at baseline of OCTAVE Sustain.

[†]Age (<30 vs ≥50) was not significant (OR, 0.46; 95% CI, 0.18–1.13; $P = 0.0910$).

[‡]Overall effect.

BID, twice daily; CI, confidence interval; CRP, C-reactive protein; EIM, extraintestinal manifestation; OR, odds ratio; PGA, Physician Global Assessment; TNFi, tumor necrosis factor inhibitor.

SUPPLEMENTARY TABLE 4. Univariate Cox Proportional Hazards Regression Analysis For Factors Associated With Time to Loss of Response During OCTAVE Sustain Among All Patients With Non-Missing Efficacy Values in OCTAVE Sustain

	**HR (95% CI)**	***P* value**
Treatment group[*]		
Tofacitinib 10 mg BID vs placebo	0.29 (0.22–0.40)	<0.0001
Tofacitinib 5 mg BID vs placebo	0.40 (0.30–0.53)	<0.0001
Albumin (g/dL; per unit)[†]	0.75 (0.56–0.99)	0.0443
PGA subscore[‡]		0.0077[‡]
1 vs 3	1.27 (0.71–2.28)	0.4222
2 vs 3	0.70 (0.54–0.91)	0.0085
Total Mayo score (per 1-point)[†]	1.12 (1.03–1.22)	0.0091
Partial Mayo score (<2 vs ≥2)[*]	0.67 (0.52–0.85)	0.0012
Age (per 10-years)[*]	0.88 (0.81–0.96)	0.0043
Age (categorical)[*,‡]		0.0375[§]
Age (<30 vs ≥50 years)	1.63 (1.17–2.28)	0.0042
EIMs at baseline of OCTAVE Induction 1 and 2 (yes vs no)[†]	1.33 (1.03–1.73)	0.0283
EIMs at baseline of OCTAVE Sustain (yes vs no)[*]	1.59 (1.11–2.30)	0.0122
CRP (mg/L) (≤6 vs >6 mg/L)[†]	0.72 (0.57–0.92)	0.0084
CRP (mg/L)[*]		
CRP (per unit)	1.02 (1.01–1.04)	0.0005
CRP (≤3 vs >3 mg/L)	0.68 (0.53–0.89)	0.0045
CRP (≤6 vs >6 mg/L)	0.59 (0.43–0.82)	0.0013

Oral corticosteroid use (yes vs no)*	2.08 (1.63–2.66)	<0.0001

Loss of response was defined by an increase in partial Mayo score of ≥2 points from baseline in OCTAVE Sustain for two consecutive visits (at least 2 weeks apart), with an increase in rectal bleeding subscore of ≥1 from baseline of OCTAVE Sustain. The following factors were not significant ($P > 0.05$): albumin (g/dL) (continuous [per unit]; <3.5 vs ≥3.5), CRP (mg/L) (continuous [per unit]; ≤3 vs >3), disease duration (<6 vs ≥6 years), endoscopic subscore, extent of disease, rectal bleeding subscore, stool frequency subscore, prior TNFi exposure (yes vs no), prior TNFi failure (yes vs no), (all at baseline of OCTAVE Induction 1 and 2); age (<65 vs ≥65 years), gender, race (Asian vs other; Black vs other; White vs other), endoscopic improvement, remission status at baseline (yes vs no), and total Mayo score (<3 vs ≥3) (all at baseline of OCTAVE Sustain).

*Data at baseline of OCTAVE Sustain.

†Data at baseline of OCTAVE Induction 1 and 2.

‡Age (30–<40 vs ≥50 years; OR, 1.35; 95% CI, 0.98–1.87; $P = 0.0666$) and (40–<50 vs ≥50 years; OR, 1.28; 95% CI, 0.91–1.80; $P = 0.1618$) were not significant.

§Overall effect.

BID, twice daily; CI, confidence interval; CRP, C-reactive protein; EIM, extraintestinal manifestation; HR, hazard ratio; PGA, Physician Global Assessment; TNFi, tumor necrosis factor inhibitor.

SUPPLEMENTARY TABLE 5. Univariate Cox Proportional Hazards Regression Analysis For Factors Associated With Time to Loss of Response During OCTAVE Sustain Among All Patients With Non-Missing Efficacy Values Who Received Tofacitinib 5 mg BID in OCTAVE Sustain

	**HR (95% CI)**	***P* value**
Total Mayo score (<3 vs ≥3)*	0.57 (0.35–0.95)	0.0302
Partial Mayo score (<2 vs ≥2)*	0.56 (0.35–0.89)	0.0150
EIMs at baseline of OCTAVE Induction 1 and 2 (yes vs no)*	1.73 (1.08–2.75)	0.0223
EIMs at baseline of OCTAVE Sustain (yes vs no)*	2.74 (1.50–4.99)	0.0010
CRP (mg/L; per unit)*	1.05 (1.00–1.11)	0.0413
Oral corticosteroid use (yes vs no)*	2.77 (1.68–4.55)	<0.0001
Albumin (g/dL)†		
Albumin (per unit)	0.52 (0.33–0.83)	0.0058
Albumin (<3.5 vs ≥3.5 g/dL)	2.86 (1.15–7.09)	0.0235
PGA subscore†		0.0035‡
1 vs 3	1.29 (0.50–3.34)	0.6056
2 vs 3	0.48 (0.30–0.78)	0.0027

Loss of response was defined by an increase in partial Mayo score of ≥2 points from baseline in OCTAVE Sustain for two consecutive visits (at least 2 weeks apart), with an increase in rectal bleeding subscore of ≥1 from baseline of OCTAVE Sustain. The following factors were not significant (*P* > 0.05): CRP (mg/L) (continuous [per unit]; ≤3 vs >3; ≤6 vs >6), disease duration (<6 vs ≥6 years), endoscopic subscore, extent of disease, rectal bleeding subscore, stool frequency subscore, prior TNFi exposure (yes vs no), prior TNFi failure (yes vs no), total Mayo score (all at baseline of OCTAVE Induction 1 and 2); age (continuous [per 10-years]; <30 vs ≥50; 30–<40 vs ≥50; 40–<50 vs ≥50; <65 vs ≥65 years), CRP (mg/L)

(≤3 vs >3; ≤6 vs >6), gender, race (Asian vs other; Black vs other; White vs other), endoscopic improvement, remission status at baseline (yes vs no) (all at baseline of OCTAVE Sustain).

[*]Data at baseline of OCTAVE Sustain.

[†]Data at baseline of OCTAVE Induction 1 and 2.

[‡]Overall effect.

BID, twice daily; CI, confidence interval; CRP, C-reactive protein; EIM, extraintestinal manifestation; HR, hazard ratio; PGA, Physician Global Assessment; TNFi, tumor necrosis factor inhibitor.

SUPPLEMENTARY TABLE 6. Univariate Cox Proportional Hazards Regression Analysis For Factors Associated With Time to Loss of Response During OCTAVE Sustain Among All Patients With Non-Missing Efficacy Values Who Received Tofacitinib 10 mg BID in OCTAVE Sustain

	**HR (95% CI)**	***P* value**
Partial Mayo score (<2 vs ≥2)*	0.53 (0.31–0.92)	0.0235
Age (per 10-years)*	0.70 (0.57–0.86)	0.0006
Age (categorical)*		0.0109†
Age (<30 vs ≥50 years)	3.73 (1.58–8.80)	0.0026
Age (30-<40 vs ≥50 years)	3.60 (1.60–8.08)	0.0019
Age (40-<50 vs ≥50 years)	2.58 (1.07–6.23)	0.0349
CRP (mg/L)*		
CRP (per unit)	1.03 (1.01–1.05)	0.0067
CRP (≤3 vs >3 mg/L)	0.50 (0.29–0.85)	0.0112
CRP (≤6 vs >6 mg/L)	0.43 (0.23–0.78)	0.0058
Oral corticosteroid use (yes vs no)*	3.28 (1.89–5.68)	<0.0001

Loss of response was defined by an increase in partial Mayo score of ≥2 points from baseline in OCTAVE Sustain for two consecutive visits (at least 2 weeks apart), with an increase in rectal bleeding subscore of ≥1 from baseline of OCTAVE Sustain. The following factors were not significant (*P* > 0.05): albumin (g/dL) (continuous [per unit]; <3.5 vs ≥3.5), C-reactive protein (CRP; mg/L) (continuous [per unit]; ≤3 vs >3; ≤6 vs >6), disease duration (<6 vs ≥6 years), presence of EIM, endoscopic subscore, extent of disease, PGA subscore, rectal bleeding subscore, prior TNFi exposure (yes vs no), prior TNFi failure (yes vs no), total Mayo score, stool frequency subscore (0 vs 3; 1 vs 3; 2 vs 3) (all at baseline of OCTAVE Induction 1 and 2); age (<65 vs ≥65 years), gender, race (Asian vs other; White vs other), presence of EIM, endoscopic improvement, remission status at baseline (yes vs no), and total Mayo score (<3 vs ≥3) (all at baseline of OCTAVE Sustain).

[*]Data at baseline of OCTAVE Sustain.

[†]Overall effect.

BID, twice daily; CI, confidence interval; CRP, C-reactive protein; EIM, extraintestinal manifestation; HR, hazard ratio; PGA, Physician Global Assessment; TNFi, tumor necrosis factor inhibitor.

SUPPLEMENTARY TABLE 7. Summary of Safety, AEs of Special Interest, and Laboratory Value Changes, By Week 52 Remission Status and Treatment Group

Remission achieved at week 52	Tofacitinib 5 mg BID		Tofacitinib 10 mg BID		Placebo	
	**Yes N = 68**	**No N = 101**	**Yes N = 80**	**No N = 83**	**Yes N = 22**	**No N = 133**
**Safety and AEs of special interest**						
AEs, n (%)	45 (66.2)	76 (75.3)	67 (83.8)	64 (77.1)	14 (63.6)	98 (73.7)
AEs leading to discontinuation, n (%)	1 (1.5)	11 (10.9)	0 (0.0)	6 (7.2)	0 (0.0)	23 (17.3)
Serious AEs, n (%)	3 (4.4)	4 (4.0)	3 (3.8)	1 (1.2)	0 (0.0)	4 (3.0)
Infections (all), n (%)	26 (38.2)	38 (37.6)	41 (51.3)	26 (31.3)	9 (40.9)	28 (21.1)
Herpes zoster (non-serious and serious), n (%)	1 (1.5)	2 (2.0)	5 (6.3)	4 (4.8)	0 (0.0)	1 (0.8)
**Laboratory value changes**						
Total cholesterol >1.3× ULN	27 (39.7)	19 (18.8)	26 (32.5)	14 (16.9)	2 (9.1)	10 (7.5)
Triglycerides >1.3× ULN	3 (4.4)	4 (4.0)	12 (15.0)	2 (2.4)	1 (4.5)	5 (3.8)
LDL >1.2× ULN	27 (39.7)	26 (25.7)	33 (41.3)	16 (19.3)	4 (18.2)	22 (16.5)

HDL <0.8× LLN	4 (5.9)	3 (3.0)	2 (2.5)	1 (1.2)	0 (0.0)	9 (6.8)
CK >2× ULN	17 (25.0)	18 (17.8)	32 (40.0)	19 (22.9)	1 (4.5)	11 (8.3)

Remission was defined as a total Mayo score ≤2 with no individual subscore >1, and a rectal bleeding subscore of 0.

AE, adverse event; BID, twice daily; CK, creatine kinase; HDL, high-density lipoprotein; LDL, low-density lipoprotein; LLN, lower limit of normal; N, number of patients in the specified category with non-missing data; n, number of patients with the specified safety event; ULN, upper limit of normal.
